# Serum Creatinine, Cystatin C and Symmetric Dimethylarginine Concentrations and Relationship Between Them in Healthy Small and Miniature Dogs: A Preliminary Study

**DOI:** 10.3390/ani15182760

**Published:** 2025-09-22

**Authors:** Julia Rafalska, Agnieszka Rusiecka, Jolanta Bujok

**Affiliations:** 1 Faculty of Veterinary Medicine, Wroclaw University of Environmental and Life Sciences, Norwida 31, 50-375 Wrocław, Poland; 2 “Wrocławska” Veterinary Clinic, Wrocławska 7, 55-040 Bielany Wrocławskie, Poland; jolanta.bujok@upwr.edu.pl; 3 Statistical Analysis Center, Biostatistics Teaching Team, Wroclaw Medical University, K. Marcinkowskiego 1, 50-368 Wrocław, Poland; agnieszka.rusiecka@umw.edu.pl; 4 Department of Animal Biostructure and Physiology, Wroclaw University of Environmental and Life Sciences, Norwida 31, 50-375 Wrocław, Poland

**Keywords:** Maltese, glomerular filtration rate, chronic kidney disease, early detection of kidney disease

## Abstract

Currently, there are common reference intervals for creatinine levels in dogs, despite different weight categories. This can result in missing the diagnosis of kidney disease at an early stage in small dogs. The aim of this study was to determine and compare available markers of the GFR (serum cystatin C, creatinine and SDMA) in healthy small and miniature dogs. Each dog underwent the same protocol, including physical examination, blood pressure measurement and ultrasound of the urinary system. The serum filtration markers determined in the study were creatinine, cystatin C and SDMA. Urinalysis was performed, including urine specific gravity (USG) and the urine protein/creatinine ratio (UPC). Forty samples were included. The median values with the IQR (interquartile range) for creatinine, SDMA and cystatin C levels in the examined dogs were 63.8 µmol/L (16.7), 10.63 µg/dL (4.64) and 0.5 mg/L (0.2), respectively. Compared with Yorkshire Terriers and Chihuahuas, Maltese dogs had significantly higher median serum creatinine concentration (*p* < 0.001). Combining results of GFR markers, USG, UPC, patient’s clinical condition as well as the breed is mandatory when assessing renal function in small dogs. Maltese dogs may have physiologically higher serum creatinine levels.

## Simple Summary

Creatinine is a marker of serum filtration and currently it is still the main parameter used to assess kidney function. As a product of muscle metabolism, it is dependent on the patient’s weight. Nevertheless, in canine veterinary medicine there are still common reference intervals for this parameter, despite differences in breed sizes. This can result in delayed diagnosis of kidney disease. The aim of the study was to assess breed, age and sex effect on renal biomarkers—serum creatinine, symmetric dimethylarginine and cystatin C—and the relationship between them in small and miniature dogs. The obtained results significantly narrow the currently used creatinine reference intervals for small dog breeds, showing that weight should always be considered when assessing kidney function in these patients, as well as other results such as physical examination, urinalysis and ultrasound of the urinary system. Additionally, creatinine levels may be physiologically higher in some breeds, as in the case of Maltese dogs.

## 1. Introduction

Assessment of kidney function in dogs is important for early diagnosis of renal disease as well as for implementation of treatment for appropriate modification of medical protocols (e.g., anesthesia) in patients with impaired kidney function. The most accurate test for assessing kidney function is the measurement of the glomerular filtration rate (GFR). GFR is essential for diagnosing and monitoring kidney diseases, as it reflects how well the kidneys are working to remove waste and excess fluid from the bloodstream. It represents the volume of fluid filtered from the glomerular capillaries into Bowman’s capsule per unit time, typically measured in milliliters per minute (mL/min/kg). The normal GFR varies depending on age, sex, body weight and breed. There have been attempts to establish a single reference interval (RI) for iohexol clearance-based GFR measurements of 3.5–4.5 mL/min/kg in healthy dogs [1], but it seems more reasonable to set separate RIs for individual weight categories. In another study, dogs were divided into body weight quartiles: 1.8–12.4, 13.2–25.5, 25.7–31.6 and 32.0–70.3 kg. RIs in different weight quartiles were 1.54–4.25, 1.29–3.50, 0.95–3.36 and 1.12–3.39 mL/min/kg, respectively [2]. Glomerular filtration rate measurement provides accurate results, but because the procedure is complicated, it is not routinely used in clinical practice. In addition, there is no proven formula for calculating the estimated GFR for dogs and cats as it is in humans. For this reason, the evaluation of kidney function in companion animals is still mainly based on the measurement of indirect markers of GFR (creatinine, symmetric dimethylarginine (SDMA) and cystatin C).

According to the International Renal Interest Society (IRIS) recommendations, the serum creatinine level in dogs should be less than 125 µmol/L [3]. Serum creatinine as a product of muscle metabolism is obviously dependent on a patient’s muscle mass. Phosphocreatine is a breakdown product in muscles, and the amount of its daily production depends on individual muscle mass. It is excreted almost exclusively by the kidneys, and its concentration in the blood is inversely proportional to the GFR [4]. In small dogs, muscle mass is markedly lower than in medium and large dogs; therefore, a small dog patient with the GFR decrease of well above 75% may still have creatinine levels within the generally accepted RI for the species. Additionally, there are significant differences in kidneys’ size; larger dogs have bigger kidneys due to the increase in size of the nephrons which can also affect serum filtration markers’ values [5,6]. The breed is also an important factor influencing creatinine concentration as greyhounds are known for their increased levels of this parameter compared to other breeds [7]. This aspect as well as the body weight of a dog was a subject in other studies, suggesting that it is still a challenge to detect kidney disease at early stages in small dogs and that there should be a separate RIs for creatinine based on both breed and body weight [8]. It is suspected that the current serum creatinine reference values are not relevant for the assessment of renal function in small and miniature dogs, as intervals were established for the general population of dogs of different body weights. This will result in a significantly delayed diagnosis of chronic kidney disease (CKD) in dogs weighing up to 5 kg as well as incorrect IRIS staging of CKD and hence, inadequate therapeutic recommendations. This is an ongoing issue which some authors have addressed and attempted to set the accurate RIs for different weight categories [9]. The diagnostic benefits of other indirect markers of GFR, such as SDMA or serum cystatin C, are currently not well understood.

Recently, SDMA measured through ELISA has been included as a complementary biomarker to serum creatinine in IRIS CKD staging guidelines. Its concentration should be lower than 18 µg/dL for nonazotemic animals (serum urea and creatinine concentrations within the RIs). SDMA is produced by the intranuclear methylation of L-arginine by arginine methyltransferase. It is released into the blood after proteolysis. It is primarily eliminated by glomerular filtration, and its concentration is not affected by reabsorption or secretion in the renal tubules [10]. There is evidence that young dogs in their growth period may present intermittently increased SDMA levels of up to 0.79 μmol/L (16 μg/dL), but no correlation between age and SDMA levels in adult dogs has been demonstrated [11]; however, more recent studies suggest that older dogs may have higher SDMA concentrations [12]. The breed of the dog may also affect the serum SDMA—greyhounds have significantly higher levels of this parameter than other breeds do; therefore, a separate RI should be used for these dogs [13]. Because SDMA is synthesized by all nucleated cells of the organism, concern was raised regarding animals with neoplasia, for which the level of this parameter would be much higher despite normal kidney function. Dogs and cats with lymphoma were shown to have increased serum SDMA levels without a simultaneous increase in serum creatinine levels [14]. Notably, changes in body fat percentage can also affect the SDMA concentration [15]. Lean body mass does not seem to affect the serum SDMA concentration [16].

Cystatin C is a cysteine protease inhibitor that is produced by all nucleated cells within the body. It is freely filtered by the glomerulus and then reabsorbed and completely decomposed, so its level in the blood correlates negatively with the GFR. Serum cystatin C is used alongside creatinine as a GFR marker in human medicine [17]. It is believed that its value does not depend on the sex, weight, height, muscle mass or age of the patient [18]. However, there was an overlap in the concentration of serum cystatin C between healthy and sick dogs (diseases other than kidney dysfunction), as well as between healthy and CKD dogs, which means that cystatin C is not currently considered an ideal marker of kidney function in animals [19].

In veterinary medicine, finding the ideal parameter for determining renal function is difficult. The problem is that there are significant differences in the overall population of dogs, such as weight or breed. Among humans, this variability is much lower, so it is easier to establish reference ranges for selected parameters. Currently, measuring creatinine levels over time in animals seems to be the most accurate way to assess renal function [20], but there is a problem when the animal that has not had blood work performed before. Some authors suggest that in small dogs weighing less than 20 kg, serum cystatin C may be useful in detecting a mild decreasing GFR [21]. The aim of this study was to determine and compare available markers of the GFR (serum cystatin C, creatinine and SDMA) in healthy dogs with a body weight of up to 5.3 kg.

## 2. Materials and Methods

### 2.1. Research Inclusion and Exclusion Criteria

Forty healthy, privately owned dogs were included in this study. The dogs had blood work and urinalysis performed before teeth scaling or castration. The main research inclusion criterion was a body weight of up to 5.3 kg. Dogs were eligible for research on the basis of a normal kidney ultrasound image, no history of acute kidney injury in the past and no symptoms that could indicate disturbed kidney function, such as polyuria and polydipsia. Besides these criteria, all the other blood work parameters were within reference intervals. Dogs were excluded from the study if azotemia was detected and/or if the urine specific gravity (USG) was low (<1.030). Some of the examined dogs had USG < 1.030; these dogs remained included if none of the other renal parameters indicated renal disease (urine protein/creatinine ratio < 0.5, clinical condition, age, history, urine sediment and ultrasound of the urinary system). Details on classification of health as well as the dog’s diet are mentioned in Supplementary Material (Tables S3 and S4, respectively). Borderline proteinuria (0.2–0.5) in the case where other parameters were normal does not yet indicate disturbed renal function, which is why they were not excluded from the research [22].

Each dog underwent the same protocol to rule out renal disease and other conditions affecting the GFR. The protocol included physical examination, muscle and body mass condition score assessment, blood pressure measurement (systemic arterial pressure (SAP) via Doppler method), ultrasound of the urinary system (using SonoScape^®^, Shenzhen, China; model P15), blood work and a complete urinalysis (using clinical refractometer and urine test strips from Meditrol^®^, Lichtenfels, Germany). Physical examination included observing and/or palpating eyes, oral cavity, ears, nose, skin, peripheral lymph nodes, abdominal cavity and limbs. Capillary refill time was measured in each dog (gingiva). Thoracic cavity was examined by auscultation. Vaginal (females), penile (males) and rectal examinations (including taking the dog’s temperature) were also performed. Hydration status was assessed in every dog. Urinalysis included tests for the presence of leukocytes, erythrocytes, glucose, nitrite, urobilinogen, ketones, bilirubin and measurement of the pH. Protein and UPC were marked in VetLab, Wrocław, Poland. Urine was collected via cystocentesis. The urine was centrifuged, 4/5 of the supernatant was discarded, the remaining part was mixed with the sediment and applied to a slide, covered with a cover slip. Ten high-power fields using the 40× objective were evaluated in a dark high-power field with a low-set condenser by an experienced person who examines urine sediment daily. Each dog had complete blood count (CBC, Sysmex^®^ XN-1000V, Hamburg, Germany) and biochemistry (Beckman coulter^®^, Indianapolis, IN, USA; model AU680/AU5800) performed to rule out any conditions affecting the GFR. The biochemical parameters included glucose, creatinine, SDMA, cystatin C, urea, alanine aminotransferase (ALT), aspartate aminotransferase (AST), alkaline phosphatase (AP), total protein (TP), albumin and globulins.

### 2.2. Glomerular Filtration Markers Assays

The levels of creatinine, SDMA, cystatin C and UPC were determined in the same commercial laboratory (VetLab, Wrocław, Poland). Assays for creatinine, SDMA and cystatin C were performed from the same serum sample by spectrophotometry: creatinine was measured by Jaffe’s method, SDMA and cystatin C were measured by immunoturbidimetry. Regarding SDMA method, immunoturbidimetry compared to LC-MS/MS (*n* = 97, r^2^ = 0.98) and IDEXX ELISA (*n* = 76, r^2^ = 0.98) showed comparable results. The laboratory reference intervals for creatinine, SDMA and cystatin C are shown in Table 1.

### 2.3. Data Analysis

The obtained data was subjected to statistical analysis using the program Statistica, version 13.3, by StatSoft, Inc., Krakow, Poland. Before proceeding to the analysis, the assumptions regarding the normality of the data distribution and homogeneity of variance were tested using the Shapiro–Wilk test and the Levene test. The quantitative data was characterized via descriptive statistics such as the arithmetic mean, standard deviation, median, quartiles and interquartile range (IQR). Appropriate tests were used in the non-parametric analysis, such as the Mann–Whitney U test for comparing two independent groups or the Kruskal–Wallis test to compare more than two independent study groups. Additionally, an analysis was conducted to assess the strength of the association between dogs’ body weight, creatinine, SDMA, cystatin C and urea levels using Spearman’s rank correlation coefficient. To identify important predictors of creatinine, SDMA and cystatin C levels in multivariate analysis, we fitted separate general linear models (GLM, Gaussian distribution, link: logit) per marker using the same set of three nominal predictors including breed, age group and sex, as well as body mass as the only continuous variable. This analysis included data from patients of the three most frequently represented breeds: Maltese, Yorkshire Terrier and Chihuahua. Differences at the level of *p* < 0.05 were considered statistically significant. All the analyzed data is available in Supplementary Material (Table S1).

## 3. Results

### 3.1. The Dogs

The study included 40 privately owned and healthy dogs whose blood work were performed before castration or teeth scaling. The dogs were between 4 and 180 months old (median 76.5) and represented the following breeds: Yorkshire Terrier (YST, *n* = 8), Maltese (*n* = 9), Chihuahua (*n* = 19), Jack Russell Terrier (*n* = 2) and mixed breed (*n* = 2). The patients weighed between 1.3 kg and 5.3 kg. The median body condition score was 5/9 (4–7), and the median muscle condition score was 3.5/4 (3–4), which is presented in Supplementary Material (Tables S6 and S7). The dogs were divided into three age groups: young dogs (<1.5 years old), adult dogs (1.5–11 years old,) and geriatric dogs (>11 years old). The dogs’ sex is presented in Table 2.

All the dogs were normotensive (median systolic blood pressure was 140 (110–160 mmHg) and did not present any abnormalities in physical examination. The ultrasound of the urinary system did not reveal any features (such as blurred renal structure, pyelectasis or presence of mineralization in the kidneys) that could indicate the impaired kidney function. Results of the CBC and urinalysis are presented in Supplementary Material (Tables S2 and S3). Table 3 contains summary statistics for dogs’ body weight, muscle condition score, body condition score as well as urine specific gravity and urine protein:creatinine ratio.

### 3.2. Correlations Between Body Weight, Sex, Age Group, Breed and Renal Function Parameters

There was no direct correlation between any of the parameters and the animal’s body weight. The creatinine level was moderately correlated with the cystatin C level (r = 0.45, *p* < 0.05), whereas no significant correlation was found between the SDMA level and the other markers. These results are contained in Table 4.

Breed was significantly correlated with obtained concentrations of all three assessed renal biomarkers (creatinine, SDMA and CysC). Sex is the valid predictor of creatinine and cystatin C concentration, while age group was an important factor affecting only the cystatin C levels (Table 5).

The levels of creatinine, cystatin C and SDMA were marked to assess kidney function in the dogs participating in the study. Obtained values are presented in Table 6.

### 3.3. Comparison of the Serum Creatinine Levels Among the Examined Dog Breeds

Creatinine levels differed significantly between dog breeds (*p* < 0.001) which is presented in Table 7. Significant differences were observed between the Maltese and Chihuahua breeds (*p* = 0.02) and the Maltese and Yorkshire Terrier breeds (*p* < 0.001). However, no significant differences were found between the Chihuahua and Yorkshire Terrier breeds (*p* = 0.92).

### 3.4. Age as a Factor Affecting Renal Function

There were no significant differences in the concentrations of the marked parameters between the age groups. The comparison between age groups is displayed in Table 8.

## 4. Discussion

Our findings support the relevance of obtaining breed specific RI as even within a population of small breed dogs (weighing up to 5.3 kg) breed variability of serum creatinine concentrations was observed. Therefore, RIs should be established not only for weight categories but also for individual breeds. In specific breeds with naturally higher creatinine levels, such as greyhounds [7] when compared with the general dog population, and Maltese when compared to other small breed dogs, simultaneous assessment of serum creatinine and other GFR markers, such as SDMA, may provide a more accurate assessment of renal function [23]. In our study there were no significant differences between males and females; however, a study conducted on a larger group suggests that males may have physiologically higher creatinine concentration than females [24]. Age could be another factor affecting the GFR as it was shown that in smaller dogs (≤15.0 kg), age was significantly and inversely correlated with plasma iohexol clearance [25], although our results from a smaller group of dogs do not indicate this.

Four out of thirty-nine dogs presented increased SDMA levels (>14 μg/dL) according to the laboratory RI, despite normal serum creatinine concentrations and no abnormalities in the other assays. Nevertheless, these dogs were not excluded from the study because the IRIS guidelines [3] and other research papers [26] suggest that using an SDMA cut-off of >18 μg/dL rather than >14 μg/dL in nonazotemic dogs increases the specificity of the assay without compromising sensitivity. These results may suggest the influence of extrarenal factors on SDMA levels in these dogs. In our study, SDMA was measured through a less commonly used immunoturbidimetry method. However, comparability with routinely used IDEXX ELISA is observed, and a similar upper reference limit (<15 µg/dL) is applicable.

Research including 18 253 human patients aged 28 to 100 years revealed that cystatin C levels were significantly higher in patients over 80 years without a risk of developing kidney disease than in patients under 40 years. These findings indicate that age affects the serum cystatin C concentration even in healthy people. Authors of the mentioned study suspect that the obtained results are consequences of the natural aging process of the kidneys, even in seemingly healthy patients [27]. In our study, there was a strong tendency (*p* = 0.18) towards increased serum cystatin C in dogs > 11 years of age, which are classified as geriatric patients [28]. Research on a 250 dogs cohort suggested that serum cystatin C concentration correlates with age but not with breed or sex [24]. Recent data shows that serum cystatin C in dogs is in fact correlated with age, weight and also significantly associated with cardiovascular or renal disease-related death [29,30]. The weight-effect is especially important because it seems that serum cystatin C is superior to serum creatinine and is a more accurate prognostic renal marker in small breed dogs (weighting up to 15–20 kg) [21]. Our findings are consistent with both human and veterinary medicine reports and support the thesis that the obtained results may be an implication of the usual loss of the GFR as the animal ages. Our results were not statistically significant (*p* > 0.05); however, GLM showed that age group was an important factor affecting the cystatin C levels (*p* = 0.005). Geriatric dogs experience inflammaging with increased inflammatory markers (immunoglobulin M) and DNA oxidation products such as 8-hydroxy-2-deoxyguanosine [31]. The chronic inflammation in these dogs may cause increased cystatin C concentration even at an early stage of kidney disease, as was discovered in elderly humans [32]. Additionally, it was shown that in obese patients, weight loss may decrease cystatin C concentration; however, authors of the mentioned study suggest that it is not correlated with improved renal function but rather with other subclinical alterations in the course of canine obesity [33].

In the case of greyhounds, factors such as a high-protein diet, intensively enlarged muscle mass and hemoconcentrated blood influence increased creatinine levels in this breed [34]. In our study, for the first time, this phenomenon was observed in Maltese dogs. It is unknown why this parameter reaches higher values in this breed than in Yorkshire Terrier and Chihuahua because the examined dogs had similar body weights and muscle condition scores and were fed commercial foods with similar compositions. This may be related to the different rates of metabolism and biochemical processes in Maltese dogs, especially in muscle tissue, which allows them to have greater absorption and/or more intense processing of creatine available in food [13]. None of the described processes have been studied in this breed, so further research is needed to understand the mechanisms of this process and to more accurately determine the biochemical profile of this breed. Additionally, not only biochemical but also the anatomical differences may influence creatinine concentration in Maltese. There were no significant differences between examined breeds, as presented in Supplementary Material in Table S5. However, the Maltese group included fewer dogs than Chihuahua and Yorkshire Terrier, so the tendency to have larger kidneys may be significant in a larger cohort. As mentioned before, larger kidneys due to the increased nephrons’ size may also affect serum filtration markers’ values [5,6].

An important diagnostic aspect is the change in the serum creatinine level during a patient’s lifetime. The median serum creatinine level in the examined chihuahua dogs was 59.2 μmol/L (IQR 19.4). If this patient experiences a significant deterioration in renal function and the creatinine level doubles, the result will still be within the accepted RI. This is the main reason why performing regular blood tests and comparing creatinine levels over time rather than using a single assay is important [20]. This issue also highlights the importance of setting specific RIs for weight and breed categories.

The main limitation of this study is the lack of direct GFR measurements to test whether every examined dog had undisturbed renal function. The GFR should be measured by calculating the renal clearance of iohexol. The gold standard is the GFR measurement via iohexol plasma purification with three blood samples. This method requires intravenous bolus injection of the marker, as well as constant monitoring of the patient [20,35]. For this reason, it is impractical for clinical use. However, an indirect GFR measurement was performed via abdominal ultrasound, muscle mass index, serum urea and creatinine levels, urine specific gravity and UPC measurements, which were sufficient to determine the renal function of the patients. Some of the blood samples were not suitable for cystatin C and SDMA assays, hence the differences in research sample sizes. To meet ASVCP reference interval guidelines criteria, this study should be extended and should include a larger cohort to improve generalizability of the results.

## 5. Conclusions

In conclusion, specific RIs should be set for different weight, breed and age categories in small dogs weighing up to 5 kg, as these factors may affect concentration of glomerular filtration markers. There are studies that confirm significant differences in serum creatinine levels depending on the patient’s weight [36,37], but there is still a lack of evidence on what the standards should be for serum creatinine in miniature breed dogs. Moreover, assessing kidney function should consider the overall results obtained (blood work, urinalysis and diagnostic imaging) and the clinical condition of the patient (body and muscle condition score, weight, hydration level and systolic blood pressure), and never based on a single parameter [38]. Cystatin C levels may be increased in geriatric dogs despite normal renal function. Finally, during evaluation of the patient’s renal function, veterinary professionals must acknowledge that serum creatinine RIs may also differ depending on the breed of the dog. Studies involving a larger population of dogs may show that Maltese dogs, similar to Greyhounds, have higher physiological serum creatinine concentrations.

## Figures and Tables

**Table 1:** The laboratory reference intervals for the chosen serum filtration parameters from VetLab Poland.

Parameter	Laboratory Reference Interval
Creatinine [μmol/L] *Creatinine [mg/dL]*	35.0–132.0 *0.40–1.53*
SDMA [μmol/L] *SDMA [μg/dL]*	0.00–0.70 *0.00–14.16*
Cystatin C [μmol/L] *Cystatin C [mg/L]*	0.60–0.65 *0.30–1.30*

**Table 2:** Sex of examined dogs.

Sex	General Group	Age Group 1	Age Group 2	Age Group 3
Male	13	1	5	7
Female	27	5	18	4

**Table 3:** Summary statistics (mean, median, quartiles) for biomarkers assessed in the study.

	General Group			Young Dogs (<1.5 Years Old)			Adult Dogs (1.5–11 Years Old)			Geriatric Dogs (>11 Years Old).		
	n	Mean *SD*	Median (Q1, Q3) *IQR*	*n*	Mean *SD*	Median (Q1, Q3) *IQR*	*n*	Mean *SD*	Median (Q1, Q3) *IQR*	*n*	Mean *SD*	Median (Q1, Q3) *IQR*
Body weight [kg]	40	3.32 *1.14*	2.9 (2.4, 4.3) *1.9*	6	2.87 *1.06*	2.45 (2.0, 4.1) *2.1*	23	3.24 1.09	2.9 (2.7, 3.9) *1.2*	11	3.75 *1.13*	4.1 (2.4, 4.8) *2.4*
Muscle condition score [scale 1–4]	40	3.65 *0.45*	4 (3, 4) *1*	6	3.42 *0.45*	3.25 (3, 4) *1*	23	3.87 *0.33*	4 (4, 4) *0*	11	3.32 *0.39*	3 (3, 3.5) *0.5*
Body condition score [scale 1–9]	40	5.45 *0.67*	5 (5, 6) *1*	6	5.33 *0.47*	5 (5, 6) *1*	23	5.48 *0.65*	5 (5, 6) *1*	11	5.45 *0.78*	5 (5, 6) *1*
Urine specific gravity	39	1.037 *0.01*	1.038 (1.028, 1.048) *0.02*	6	1.048 *0.01*	1.053 (1.038, 1.056) *0.018*	22	1.037 *0.01*	1.039 (1.036, 1.044) *0.008*	11	1.029 *0.01*	1.025 (1.020, 1.045) *0.025*
Urine protein: creatinine ratio	39	0.20 *0.13*	0.14 (0.115, 0.252) *0.137*	6	0.191 *0.06*	0.177 (0.13, 0.265) *0.135*	22	0.174 *0.11*	0.126 (0.110, 0.209) *0.099*	11	0.259 *0.18*	0.159 (0.122, 0.427) *0.305*

n—number of dogs. Q1—the first quartile. Q3—the third quartile. IQR—interquartile range.

**Table 4:** Spearman’s rank correlation coefficient between dogs’ body weight, serum creatinine, serum SDMA, serum cystatin C and urea levels.

Variable	Serum SDMA	Serum Cystatin C	Body Weight	Serum Urea	Serum Creatinine
Serum SDMA	1.000000	0.167542	−0.173852	0.244576	0.240421
Serum Cystatin C	0.167542	1.000000	−0.016328	0.195697	0.446502 *
Body Weight	−0.173852	−0.016328	1.000000	−0.242231	0.216383
Serum Urea	0.244576	0.195697	−0.242231	1.000000	0.150588
Serum Creatinine	0.240421	0.446502 *	0.216383	0.150588	1.000000

* *p* < 0.05.

**Table 5:** Summary of model components. Degrees of freedom (df), Wald’s statistics, and *p*-values are given for each independent GLM model predicting creatinine, SDMA and cystatin C levels.

		Creatinine [µmol/L]		SDMA [µg/dL]		Cystatin C [mg/L]	
Factor	df	Wald’s Statistics	*p*-Value	Wald’s Statistics	*p*-Value	Wald’s Statistics	*p*-Value
Intercept	1	1707,635	0.000000	245.2578	0.000000	19.87223	0.000008
Body weight [kg]	1	0.076	0.782933	0.8840	0.347105	0.49802	0.480373
Sex	1	6.102	0.013501	0.1046	0.949024	18.47629	0.000097
Age group	2	1.663	0.435447	0.0949	0.758082	7.81716	0.005175
Breed	2	18.980	0.000076	8.5479	0.013927	9.43862	0.008921

**Table 6:** Summary statistics (mean, median, quartiles, minimum, maximum) for determined renal parameters: creatinine, cystatin C and SDMA in examined dogs.

Serum Filtration Parameters in the Examined Dogs	n	Mean *SD*	Median (Q1, Q3) *IQR*	Minimum	Maximum
Creatinine [µmol/L]	40	65.1 *13.56*	63.8 (57.1, 73.8) *22.1*	44.2	96.0
Cystatin C [mg/L]	37	0.56 *0.11*	0.5 (0.5, 0.7) *0.2*	0.30	0.70
SDMA [µg/dL]	39	10.48 *2.84*	10.63 (7.9, 12.5) *4.6*	5.86	17.09

n—number of dogs. Q1—the first quartile. Q3—the third quartile. IQR—interquartile range.

**Table 7:** Summary statistics (mean, median, quartiles, minimum, maximum) and results of Kruskal–Wallis tests for serum concentrations of cystatin C, SDMA and creatinine across the dogs’ breeds.

Creatinine [µmol/L]	n	Mean *SD*	Minimum	Maximum	Median (Q1, Q3) *IQR*	*p*-Value		
Yorkshire Terrier	8	56.3 *10.66*	44.2	72.6	59.2 (44.7, 62.9) *18.2*	0.92		<0.001
Maltese	9	76.9 *12.59*	62.1	96.0	76.4 (66.9, 83.8) *16.9*		0.02	
Chihuahua	19	61.7 *11.07*	44.9	80.7	60.8 (53.3, 72.7) *19.4*			

n—number of dogs. Q1—the first quartile. Q3—the third quartile. IQR—interquartile range.

**Table 8:** Summary statistics (mean, median, quartiles) and results of Kruskal–Wallis tests for serum concentrations of cystatin C, SDMA and creatinine across the age groups.

	Young Dogs (<1.5 Years Old)			Adult Dogs (1.5–11 Years Old)			Geriatric Dogs (>11 Years Old)			
	n	Mean *SD*	Median (Q1, Q3) *IQR*	*n*	Mean *SD*	Median (Q1, Q3) *IQR*	*n*	Mean *SD*	Median (Q1, Q3) *IQR*	*p*-Value
Cystatin C [mg/L]	6	0.53 *0.10*	0.5 (0.5, 0.6) *0.1*	22	0.55 *0.11*	0.5 (0.5, 0.7) *0.2*	9	0.62 *0.11*	0.7 (0.6, 0.7) *0.1*	0.18
SDMA [µg/dL]	6	10.56 *3.16*	10.97 (8.42, 13.07) *4.65*	23	10.74 *2.99*	10.85 (7.86, 12.67) *4.81*	10	9.83 *2.45*	9.78 (7.90, 12.04) *4.14*	0.71
Creatinine [μmol/L]	6	61.65 *5.76*	61.4 (57.6, 64.8) *7.2*	23	65.88 *13.12*	64.9 (57.0, 76.4) *19.4*	11	66.34 *17.56*	64.5 (50.3, 77.3) *27.0*	0.79

n—number of dogs. Q1—the first quartile. Q3—the third quartile. IQR—interquartile range.

## Data Availability

The original contributions presented in this study are included in the Supplementary Material. Further inquiries can be directed to the corresponding author.

## Supplementary Materials

The following supporting information can be downloaded at https://www.mdpi.com/article/10.3390/ani15182760/s1. Table S1. Original Data obtained from examined dogs used in statistical analysis in the study. Table S2. Complete blood count of examined dogs. Table S3. Urinalysis results of the examined dogs. Table S4. Diet of the examined dogs. Table S5. Kidneys’ sizes of examined dogs. The kidneys were measured via abdominal ultrasound. Table S6. Blood pressure of examined dogs. Table S7. Dogs’ body and muscle condition score.
