# Supplementary material for: Serum Creatinine, Cystatin C and Symmetric Dimethylarginine Concentrations and Relationship Between Them in Healthy Small and Miniature Dogs: A Preliminary Study
Source: Animals (Basel). 2025 Sep 22;15(18):2760. doi: 10.3390/ani15182760 (PMC12466481; doi:10.3390/ani15182760)
Supplement: Supplementary file 1 [file animals-15-02760-s001.zip › animals-3689403-supplementary.pdf]

Supplementary Material

Supplementary Tables:

Patient's ID	Breed	Age [months]	Age group	Sex	Body weight [kg]	Urea [mmol/l]	Creatinine [µmol/l]	SDMA [µg/dl]	Cystatin C [mg/l]
D1	Chihuahua	18	1	Female	4.1	6.69	64.8	5.86	0.60
D2	Chihuahua	15	1	Male	2.6	3.69	57.6	8.42	0.50
D3	Chihuahua	7	1	Female	1.7	5.25	63.1	9.49	0.50
D4	Chihuahua	7	1	Female	2.0	4.65	54.2	12.44	0.50
D5	Mix-breed (cavapoo)	12	1	Female	4.5	6.50	70.5	13.07	0.40
D6	Yorkshire Terrier	4	1	Female	2.3	7.65	59.7	14.06	0.70
D7	Chihuahua	30	2	Male	2.3	7.65	72.7	13.61	0.50
D8	Chihuahua	24	2	Female	2.7	4.00	50.2	7.66	0.40
D9	Chihuahua	78	2	Female	3.1	5.08	77.8	7.03	0.50
D10	Chihuahua	66	2	Female	2.7	5.85	80.7	10.95	0.70
D11	Chihuahua	60	2	Female	2.7	5.13	66.1	8.10	0.50
D12	Chihuahua	39	2	Female	1.3	6.99	60.8	7.86	0.50
D13	Chihuahua	108	2	Male	2.3	9.12	53.3	10.85	0.50
D14	Chihuahua	88	2	Female	2.9	5.28	45.2	7.62	-
D15	Chihuahua	108	2	Female	4.3	3.36	57.0	9.98	0.50
D16	Jack Russell Terrier	66	2	Male	6.1	3.91	62.4	8.71	0.50
D17	Maltese	80	2	Female	3.0	3.49	67.1	6.32	0.70
D18	Maltese	48	2	Female	2.1	7.61	92.7	10.93	0.70
D19	Maltese	35	2	Female	5.6	5.17	82.9	12.34	0.70
D20	Maltese	72	2	Female	3.6	4.16	66.9	12.34	0.50
D21	Maltese	18	2	Female	2.9	7.54	76.4	11.33	0.50
D22	Maltese	54	2	Male	3.9	4.83	62.1	17.09	0.50
D23	Maltese	78	2	Female	4.6	4.23	83.8	14.95	0.50
D24	Yorkshire Terrier	81	2	Male	4.3	2.07	44.2	7.51	0.30
D25	Yorkshire Terrier	76	2	Female	3.3	4.85	64.9	13.88	0.70

D26	Yorkshire Terrier	36	2	Female	2.7	7.66	60.9	10.08	0.50
D27	Yorkshire Terrier	36	2	Female	2.7	7.11	72.6	15.86	0.70
D28	Yorkshire Terrier	77	2	Female	2.7	6.70	58.6	9.37	0.60
D29	Yorkshire Terrier	103	2	Female	2.7	5.71	45.1	12.67	0.50
D30	Chihuahua	126	3	Female	3.8	4.37	77.3	8.06	0.70
D31	Chihuahua	136	3	Male	5.0	5.89	57.2	7.39	0.60
D32	Chihuahua	146	3	Female	2.3	5.06	75.0	10.63	0.70
D33	Chihuahua	180	3	Female	2.4	13.80	64.9	13.64	-
D34	Chihuahua	135	3	Male	4.4	6.35	44.9	7.90	0.40
D35	Chihuahua	168	3	Male	2.2	8.89	50.3	-	-
D36	Jack Russell Terrier	156	3	Male	4.8	8.05	92.8	12.54	0.60
D37	Maltese	144	3	Male	4.1	8.78	96.0	8.93	0.70
D38	Maltese	156	3	Male	5.0	11.80	64.5	10.77	0.70
D39	Mix-breed	129	3	Female	4.8	6.52	62.6	6.36	0.50
D40	Yorkshire Terrier	122	3	Male	2.4	4.32	44.2	12.04	0.70

Table S1. Original Data obtained from examined dogs used in statistical analysis in the study.
* Dogs were divided into three age groups: 1. young dogs (<1.5 years old), 2. adult dogs (1.5-11 years old) and 3. geriatric dogs (>11 years old).

Patient's ID	White blood cells [G/l]	Neutrophils [G/l]	Neutrophils [%]	Lympho-cytes [G/l]	Lympho-cytes [%]	Mono-cytes [G/l]	Mono-cytes [%]	Eosino phils [G/l]	Eosino-phils [%]	Baso-phils [G/l]	Baso-phils [%]	Red blood cells [T/l]	Hemo-globin [g/l]	Hemato -crit fraction [l/l]	Mean cell volume [fl]	Mean corpu-scular hemo-globin mass [pg]	Mean corpu-scular hemo-globin concent-ration [g/l]	Platelets [G/l]
D1	11.30	5.29	47.0	4.58	40.70	0.71	6.30	0.64	5.70	0.03	0.30	7.15	182.0	0.54	74.80	25.50	340.0	246.0
D2	9.62	5.84	60.70	2.70	28.10	0.45	4.70	0.58	6.0	0.05	0.50	8.27	195.0	0.55	66.30	23.60	356.0	172.0
D3	12.10	6.36	52.90	4.62	38.30	0.74	6.10	0.29	2.40	0.04	0.30	6.34	159.0	0.46	72.90	25.10	344.0	304.0
D4	12.30	9.16	74.20	2.19	17.70	0.79	6.40	0.18	1.50	0.02	0.20	5.93	142.0	0.41	69.80	23.90	343.0	653.0
D5	12.20	6.51	53.30	4.55	37.30	0.73	6.0	0.36	3.0	0.05	0.40	6.57	158.0	0.47	71.20	24.0	338.0	91.0
D6	8.39	4.32	51.50	3.24	38.60	0.57	6.80	0.21	2.50	0.05	0.60	6.78	160.0	0.46	68.30	23.60	246.0	200.0
D7	7.66	3.58	46.70	3.02	39.40	0.55	7.20	0.49	6.40	0.02	0.30	8.39	200.0	0.57	67.90	23.80	351.0	304.0
D8	8.79	6.33	72.0	1.66	18.90	0.50	5.70	0.29	3.30	0.01	0.10	7.13	177.0	0.49	69.10	24.80	359.0	270.0
D9	11.20	8.56	76.60	1.96	17.50	0.43	3.80	0.22	2.0	0.01	0.10	7.91	206.0	0.57	71.70	26.0	363.0	371.0
D10	8.49	5.38	63.40	2.29	27.0	0.42	4.90	0.39	4.60	0.01	0.10	7.77	209.0	0.56	71.60	26.90	376.0	291.0
D11	8.44	5.83	69.10	1.32	15.60	0.48	5.70	0.77	9.10	0.04	0.50	6.55	168.0	0.48	73.30	25.60	350.0	184.0
D12	11.60	6.68	57.80	3.57	30.90	0.77	6.70	0.51	4.40	0.02	0.20	6.32	156.0	0.44	70.10	24.70	352.0	279.0
D13	10.30	6.03	58.70	2.57	25.0	0.80	7.80	0.82	8.0	0.05	0.50	7.19	159.0	0.48	67.0	22.10	330.0	277.0
D14	8.80	5.69	64.70	2.13	24.20	0.50	5.70	0.46	5.20	0.02	0.20	6.76	161.0	0.47	69.10	23.80	345.0	380.0
D15	12.20	6.65	54.50	4.28	35.20	0.75	6.20	0.47	3.90	0.02	0.20	6.23	149.9	0.43	68.20	23.90	351.0	325.0
D16	10.90	7.40	67.90	2.45	22.50	0.42	3.80	0.57	5.20	0.07	0.60	6.61	157.0	0.46	69.70	23.80	341.0	213.0
D17	5.63	2.66	47.20	2.13	37.80	0.64	11.40	0.15	2.70	0.05	0.90	7.43	181.0	0.52	70.10	24.40	347.0	213.0
D18	6.14	4.23	65.90	1.16	18.10	0.71	11.10	0.30	4.70	0.01	0.20	5.29	141.0	0.40	75.20	26.70	354.0	580.0
D19	10.90	7.10	64.90	2.90	26.50	0.52	4.80	0.39	3.60	0.02	0.20	7.86	182.0	0.52	66.20	23.20	350.0	244.0

D20	8.76	5.40	61.70	2.69	30.70	0.38	4.30	0.28	3.20	0.01	0.10	7.95	206.0	0.56	70.60	25.90	367.0	382.0
D21	10.50	5.83	55.80	3.78	36.20	0.42	4.0	0.41	3.90	0.01	0.10	6.42	175.0	0.49	76.0	27.30	359.0	258.0
D22	16.45	11.04	67.20	3.44	20.90	1.39	8.40	0.56	3.40	0.02	0.10	6.64	165.0	0.47	70.80	24.80	351.0	324.0
D23	6.14	4.01	65.30	1.56	25.40	0.38	6.20	0.18	2.90	0.01	0.20	6.65	182.0	0.48	72.50	27.40	378.0	566.0
D24	11.0	7.86	71.40	2.23	20.20	0.61	5.50	0.31	2.80	0.01	0.10	7.29	149.0	0.44	60.20	20.40	339.0	90.0
D25	6.75	3.77	55.80	2.26	33.50	0.44	6.50	0.24	3.60	0.04	0.60	7.49	187.0	0.52	69.80	25.0	358.0	228.0
D26	8.51	4.52	53.10	3.50	41.10	0.35	4.10	0.11	1.30	0.03	0.40	7.21	182.0	0.51	70.0	25.20	360.0	167.0
D27	9.14	4.84	53.0	3.28	35.90	0.60	6.60	0.37	4.0	0.05	0.50	7.89	197.0	0.55	69.30	25.0	360.0	269.0
D28	11.34	6.51	57.40	3.49	30.80	1.02	9.0	0.30	2.60	0.02	0.20	8.47	183.0	0.55	64.30	21.60	336.0	343.0
D29	11.89	7.07	59.50	3.94	33.20	0.63	5.30	0.18	1.50	0.07	0.60	7.77	169.0	0.48	62.0	21.70	353.0	322.0
D30	9.79	6.23	63.70	2.55	26.0	0.37	3.80	0.61	6.20	0.03	0.30	8.13	213.0	0.58	71.10	26.20	369.0	233.0
D31	15.20	11.40	74.80	2.31	15.20	0.92	6.10	0.58	3.80	0.01	0.10	7.52	196.0	0.55	73.70	26.10	354.0	149.0
D32	10.80	7.61	70.80	1.95	18.10	0.68	6.30	0.49	4.60	0.02	0.20	6.21	147.0	0.43	68.90	23.70	343.0	232.0
D33	7.11	4.24	59.70	1.93	27.10	0.69	9.70	0.25	3.50	0.0	0.0	6.23	147.0	0.42	67.90	23.60	348.0	526.0
D34	15.70	11.30	71.50	3.10	19.70	1.13	7.20	0.24	1.50	0.02	0.10	6.10	150.0	0.44	72.0	24.60	342.0	353.0
D35	15.30	9.55	62.60	3.90	25.60	0.76	5.0	1.01	6.60	0.03	0.20	6.38	163.0	0.47	73.0	25.50	350.0	337.0
D36	5.18	3.43	66.20	1.18	22.80	0.33	6.40	0.22	4.20	0.02	0.40	6.56	158.0	0.44	66.80	24.10	361.0	529.0
D37	6.53	4.26	65.20	1.62	24.80	0.49	7.50	0.16	2.50	0.0	0.0	7.05	178.0	0.51	71.90	25.20	351.0	357.0
D38	10.70	6.39	59.90	2.48	23.30	1.12	10.50	0.64	6.0	0.03	0.30	5.98	148.0	0.42	70.90	24.70	349.0	540.0
D39	6.02	4.56	75.80	1.11	18.40	0.20	3.30	0.15	2.50	0.0	0.0	6.33	156.0	0.48	76.0	24.6	324.0	93.0
D40	5.93	4.39	73.90	1.26	21.30	0.26	4.40	0.02	0.30	0.0	0.10	8.79	202.0	0.59	67.0	23.0	343.0	207.0

Table S2. Complete blood count of examined dogs.

Patient's ID	Blood [Ery/µl]	Leukocytes [Leu/µl]	Urobilinogen [µmol/L]	Bilirubin	Nitrite	Ketones	Glucose (mmol/L)	pH	Protein [g/L]	Urine Specific Gravity	Urine Protein: Creatinine Ratio	Urine sediment	Comments
D1	negative	negative	negative	negative	negative	negative	negative	6.0	0.31	1.056	0.130	Inactive	
D2	ca. 10	negative	negative	positive	negative	negative	negative	5.5	0.27	1.030	0.265	Inactive with multiple bilirubin crystals	Few erythrocytes may occur in urine due to cystocentesis. Healthy dogs may excrete bilirubin into the urine since they have low bilirubin renal threshold.
D3	negative	negative	negative	negative	negative	negative	negative	6.0	0.53	1.052	0.184	Inactive	
D4	negative	negative	negative	positive	negative	negative	negative	7.0	0.09	1.038	0.169	Inactive	Healthy dogs may excrete bilirubin into the urine since they have low bilirubin renal threshold.
D5	negative	negative	negative	negative	negative	negative	negative	6.5	0.23	1.054	0.130	Inactive	
D6	negative	negative	negative	positive	negative	negative	negative	5.5	0.43	1.058	0.269	Inactive	Healthy dogs may excrete bilirubin into the urine since they have low bilirubin renal threshold.
D7	negative	negative	negative	negative	negative	negative	negative	5.0	0.26	1.039	0.140	Inactive	
D8	negative	negative	negative	positive	negative	negative	negative	5.5	0.28	1.042	0.115	Inactive	Healthy dogs may excrete bilirubin into the urine since they have low

													bilirubin renal threshold.
D9	ca. 50	negative	negative	positive	negative	negative	negative	5.5	0.45	1.041	0.209	Inactive	Few erythrocytes may occur in urine due to cystocentesis. Healthy dogs may excrete bilirubin into the urine since they have low bilirubin renal threshold.
D10	negative	negative	negative	positive	negative	negative	negative	6.5	0.07	1.015	0.115	Inactive	Not the first urine of the day & fed mainly a wet diet. Healthy dogs may excrete bilirubin into the urine since they have low bilirubin renal threshold.
D11	negative	negative	negative	negative	negative	negative	negative	6.0	0.65	1.055	0.267	Inactive	
D12	negative	negative	negative	positive	negative	negative	negative	5.5	0.24	1.056	0.105	Inactive	Healthy dogs may excrete bilirubin into the urine since they have low bilirubin renal threshold.
D13	negative	ca. 25	negative	positive	negative	negative	negative	5.0	0.13	1.013	0.497	Inactive	Not the first urine of the day & fed only a wet diet. Healthy dogs may excrete bilirubin into the urine since they have low bilirubin renal threshold.

D14	negative	negative	negative	positive	negative	negative	negative	6.0	0.19	1.039	0.252	0-1 leukocytes per high-power field	Healthy dogs may excrete bilirubin into the urine since they have low bilirubin renal threshold. Up to 5 WBCs per HPF may be present in healthy animals.
D15	negative	ca. 25	negative	positive	negative	negative	negative	5.5	0.20	1.036	0.113	0-1 leukocytes per high-power field	Healthy dogs may excrete bilirubin into the urine since they have low bilirubin renal threshold. Up to 5 WBCs per HPF may be present in healthy animals.
D16	negative	ca. 25	negative	negative	negative	negative	negative	7.0	0.03	1.007	0.078	Inactive	Fed only a wet and homemade diet.
D17	negative	ca. 50	negative	negative	negative	negative	negative	6.0	0.31	1.030	0.164	Inactive	
D18	negative	ca. 25	negative	negative	negative	negative	negative	6.5	0.18	1.036	0.067	Inactive	
D19	negative	negative	negative	negative	negative	negative	negative	6.5	0.31	1.044	0.094	Inactive	
D20	negative	ca. 25	negative	positive	negative	negative	negative	5.5	0.13	1.032	0.123	Inactive	Healthy dogs may excrete bilirubin into the urine since they have low bilirubin renal threshold.
D21	negative	ca. 25	negative	positive	negative	negative	negative	6.0	0.09	1.036	0.091	1-4 erythrocytes per high-power field	Few erythrocytes may occur in urine due to cystocentesis. Healthy dogs may excrete bilirubin into the urine since

													they have low bilirubin renal threshold.
D22	negative	ca. 25	negative	positive	negative	negative	negative	6.5	0.72	1.038	0.469	Inactive	Few erythrocytes may occur in urine due to cystocentesis. Healthy dogs may excrete bilirubin into the urine since they have low bilirubin renal threshold.
D23	-	-	-	-	-	-	-	-	-	-	-	-	
D24	negative	negative	negative	positive	negative	negative	negative	6.0	0.23	1.038	0.110	Inactive	Healthy dogs may excrete bilirubin into the urine since they have low bilirubin renal threshold.
D25	negative	negative	negative	negative	negative	negative	negative	5.0	0.30	1.048	0.160	Inactive	
D26	ca. 50	negative	negative	positive	negative	negative	negative	5.0	0.24	1.049	0.129	Inactive	Few erythrocytes may occur in urine due to cystocentesis. Healthy dogs may excrete bilirubin into the urine since they have low bilirubin renal threshold.
D27	negative	negative	negative	positive	negative	negative	negative	5.5	0.22	1.042	0.116	Inactive	Healthy dogs may excrete bilirubin into the urine since they have low

													bilirubin renal threshold.
D28	negative	negative	negative	negative	negative	negative	negative	6.5	0.35	1.058	0.219	Inactive	
D29	negative	negative	negative	positive	negative	negative	negative	7.0	0.22	1.037	0.205	Inactive	Healthy dogs may excrete bilirubin into the urine since they have low bilirubin renal threshold.
D30	ca. 25	negative	negative	negative	negative	negative	negative	6.5	0.09	1.011	0.133	Inactive	Collecting the morning urine was impossible because the patient urinates on pads at home.
D31	negative	negative	negative	negative	negative	negative	negative	6.0	0.41	1.045	0.140	Inactive	
D32	negative	negative	negative	positive	negative	negative	negative	5.5	0.57	1.021	0.427	Inactive	Not the first urine of the day. Healthy dogs may excrete bilirubin into the urine since they have low bilirubin renal threshold.
D33	negative	negative	negative	positive	negative	negative	negative	5.5	0.34	1.030	0.596	0-1 leukocytes per high-power field	Not the first urine of the day & fed only a wet diet. Healthy dogs may excrete bilirubin into the urine since they have low bilirubin renal threshold. Up to 5 WBCs per HPF may be present in healthy animals.

D34	negative	negative	negative	negative	negative	negative	negative	6.0	0.18	1.028	0.293	Inactive	Fed only a wet diet. Healthy dogs may excrete bilirubin into the urine since they have low bilirubin renal threshold.
D35	negative	negative	negative	positive	negative	negative	negative	5.5	0.07	1.025	0.245	Inactive	Fed only a wet diet. Healthy dogs may excrete bilirubin into the urine since they have low bilirubin renal threshold.
D36	negative	negative	negative	positive	negative	negative	negative	6.5	0.08	1.020	0.090	3-5 leukocytes per high-power field, multiple sperm cells	Not the first urine of the day & fed only a wet diet. Healthy dogs may excrete bilirubin into the urine since they have low bilirubin renal threshold. Up to 5 WBCs per HPF may be present in healthy animals.
D37	ca. 50	ca. 25	negative	negative	negative	negative	negative	7.0	0.07	1.020	0.080	0-1 erythrocytes per high-power field	Fed mainly a wet diet. Few erythrocytes may occur in urine due to cystocentesis.
D38	negative	negative	negative	positive	negative	negative	negative	5.0	0.99	1.047	0.573	Multiple sperm cells	Healthy dogs may excrete bilirubin into the urine since they have low

													bilirubin renal threshold.
D39	negative	negative	negative	negative	negative	negative	negative	5.0	0.24	1.052	0.122	0-1 leukocytes per high-power field	Up to 5 WBCs per HPF may be present in healthy animals.
D40	negative	ca. 25	negative	positive	negative	negative	negative	6.5	0.26	1.025	0.159	Inactive	Not the first urine of the day & fed mainly a wet diet. Healthy dogs may excrete bilirubin into the urine since they have low bilirubin renal threshold.

Table S3. Urinalysis results of the examined dogs.
Parameters marked yellow were assayed using urine test strips (Meditrol®).
The patients marked blue were re-checked due to proteinuria/ low urine specific gravity.
HPF- high-power field

Patient's ID	Diet	Type of food
D1	VetExpert Raw Paleo® Beef	Dry and wet
D2	VetExpert Raw Paleo® Turkey	Dry and wet
D3	Dolina Noteci® Guinea Fowl	Dry
D4	MeatLove® & Wiejska Zagroda® *	Wet
D5	Royal Canin® Mini Adult	Dry

D6	Royal Canin® Yorkshire Terrier Puppy	Dry
D7	Royal Canin® Chihuahua Adult	Wet
D8	Psi Bufet® Turkey & Beef	Wet
D9	Raw Paleo® Turkey & Homemade meals (turkey, rice, carrot)	Dry and wet
D10	MeatLove®* & Farmina N&D® Boar	Dry and wet
D11	Raw Paleo® Turkey & Homemade meals (turkey, rice, carrot)	Dry and wet
D12	Raw Paleo® Turkey & Homemade meals (turkey, rice, carrot)	Dry and wet
D13	Dolina Noteci®* & Bemo®*	Wet
D14	Dolina Noteci®* & Bemo®*	Wet
D15	Dolina Noteci®* & Bemo®*	Wet
D16	John Dog® Lamb and Beef & Homemade meals (beets, carrots, sweet potatoes, pears, apples, yoghurt, eggs)	Wet
D17	Brit Care® Grain-free Adult Small and Medium Salmon & Dolina Noteci®	Dry and wet
D18	Syta Micha® Beef with Carrot and Apple & Rabbit with Vegetables & Rabbit with Swede, Strawberry and Rosemary	Wet
D19	Royal Canin® Maltese Adult	Dry
D20	Psiastki® Duck and Pear	Wet
D21	Psiastki® Duck and Pear	Wet
D22	Psiastki® Duck and Pear	Wet

D23	Animonda GranCarno® Beef	Wet
D24	Hill´s Prescription Diet® Canine u/d Non-Struvite Urinary	Steeped dry food
D25	Royal Canin® Yorkshire Terrier Adult	Dry
D26	Royal Canin® Yorkshire Terrier Adult	Dry
D27	Royal Canin® Yorkshire Terrier Adult	Dry
D28	Royal Canin® Yorkshire Terrier Adult	Dry
D29	Dolina Noteci®*	Dry and wet
D30	Royal Canin® Chihuahua Adult	Wet
D31	ORIJEN® Small Breed	Dry
D32	Vet Expert® Renal Elimination Dog	Dry
D33	Raw Paleo® Turkey & Homemade meals (turkey, rice, carrot)	Wet
D34	Raw Paleo® Turkey & Homemade meals (turkey, rice, carrot)	Wet
D35	Raw Paleo® Turkey & Homemade meals (turkey, rice, carrot)	Wet
D36	Farmina N&D® Prime Boar & Apple	Wet
D37	Wiejska Zagroda® Lamb and Sweet Potatoes & Dogs Plate®	Dry and wet
D38	Applaws® Dog Small & Medium Breed Adult Chicken	Dry
D39	Hill's Prescription Diet® Metabolic + Mobility Chicken	Dry
D40	Dolina Noteci®*	Dry and wet

Table S4. Diet of the examined dogs.
* Patient was fed with more than one kind of food from the indicated company.

Patient's ID	Breed	Sex	Left kidney [length & width, cm]	Right kidney [length & width, cm]
D1	Chihuahua	Female	3.68 x 1.88	3.62 x 1.96
D2	Chihuahua	Male	3.85 x 1.90	4.0 x 3.80
D3	Chihuahua	Female	2.84 x 1.66	2.99 x 17.1
D4	Chihuahua	Female	2.91 x 1.60	3.15 x 1.63
D5	Mix-breed (cavapoo)	Female	3.74 x 1.99	3.77 x 1.88
D6	Yorkshire Terrier	Female	2.80 x 1.50	2.96 x 1.50
D7	Chihuahua	Male	2.88 x 1.49	3.19 x 1.49
D8	Chihuahua	Female	3.40 x 1.73	3.12 x 1.96
D9	Chihuahua	Female	3.07 x 1.61	3.45 x 1.80
D10	Chihuahua	Female	2.79 x 2.20	3.28 x 1.65
D11	Chihuahua	Female	2.50 x 1.45	3.15 x 1.50
D12	Chihuahua	Female	2.12 x 1.38	2.59 x 1.46
D13	Chihuahua	Male	3.26 x 1.79	3.26 x 1.79
D14	Chihuahua	Female	3.45 x 1.91	3.45 x 1.91
D15	Chihuahua	Female	3.57 x 2.01	3.84 x 2.03
D16	Jack Russell Terrier	Male	3.66 x 2.03	3.96 x 2.06
D17	Maltese	Female	2.97 x 1.61	2.97 x 1.61
D18	Maltese	Female	2.85 x 1.55	3.10 x 1.60
D19	Maltese	Female	3.79 x 4.0	4.0 x 3.80

D20	Maltese	Female	3.45 x 1.90	3.34 x 1.60
D21	Maltese	Female	2.15 x 1.34	3.94 x 1.83
D22	Maltese	Male	3.92 x 1.99	4.27 x 1.92
D23	Maltese	Female	3.60 x 1.90	3.75 x 1.85
D24	Yorkshire Terrier	Male	3.82 x 3.05	4.18 x 3.09
D25	Yorkshire Terrier	Female	3.36 x 2.0	3.55 x 2.2
D26	Yorkshire Terrier	Female	3.16 x 1.41	3.21 x 1.53
D27	Yorkshire Terrier	Female	2.80 x 1.60	3.18 x 1.70
D28	Yorkshire Terrier	Female	3.16 x 1.86	3.35 x 1.77
D29	Yorkshire Terrier	Female	3.32 x 1.81	3.39 x 1.83
D30	Chihuahua	Female	3.06 x 1.76	3.39 x 1.62
D31	Chihuahua	Male	3.60 x 1.90	3.20 x 1.90
D32	Chihuahua	Female	3.73 x 1.81	3.46 x 1.80
D33	Chihuahua	Female	2.77 x 1.82	2.89 x 1.60
D34	Chihuahua	Male	4.47 x 2.16	4.0 x 2.22
D35	Chihuahua	Male	3.02 x 1.75	2.81 x 1.70
D36	Jack Russell Terrier	Male	4.46 x 2.39	4.29 x 2.35
D37	Maltese	Male	3.45 x 2.25	3.67 x 2.10
D38	Maltese	Male	4.33 x 2.06	4.62 x 2.47
D39	Mix-breed	Female	3.50 x 1.97	3.90 x 2.0
D40	Yorkshire Terrier	Male	3.18 x 1.45	3.18 x 1.45

Table S5. Kidneys' sizes of examined dogs. The kidneys were measured via abdominal ultrasound.

Patient's ID	Blood pressure [mmHg]	Comments

D1	125	
D2	140	
D3	135	
D4	150	
D5	120	
D6	120	
D7	140	
D8	135	
D9	135	
D10	125	
D11	155	Very stressed, hyper ventilation during physical exam
D12	125	
D13	140	
D14	125	
D15	110	
D16	155	
D17	160	Very stressed, hyper ventilation during physical exam
D18	150	
D19	160	Very stressed, hyper ventilation during physical exam
D20	140	
D21	155	
D22	145	

D23	160	Very stressed-first time in the clinic. Due to the high value of the first measurement (180), examination was repeated and the presented value is a mean from 3 measurements taken 15 minutes between each other. (180,150,150 mmHg)
D24	130	
D25	150	
D26	125	
D27	140	
D28	135	
D29	135	
D30	155	
D31	155	
D32	125	
D33	110	
D34	160	
D35	160	
D36	130	
D37	155	
D38	150	
D39	130	
D40	125	
**Median**	**140**	
**Q1**	**125**	
**Q3**	**155**	
**IQR**	**30**	

Table S6. Blood pressure of examined dogs. Q1- the first quartile, Q3- the third quartile, IQR- interquartile range

Patient's ID	Muscle Condition Score [1-4]	Body Condition Score [1-9]
D1	4	6
D2	3.5	6
D3	3	5
D4	3	5
D5	3	5
D6	4	5
D7	4	5
D8	4	6
D9	4	6
D10	4	6
D11	4	6
D12	4	5
D13	3	5
D14	4	5
D15	4	7
D16	4	5
D17	4	6
D18	4	6
D19	4	6
D20	3	4
D21	4	5
D22	4	5
D23	4	6
D24	4	5

D25	4	6
D26	4	5
D27	4	5
D28	4	5
D29	3	6
D30	4	6
D31	3.5	6
D32	3	6
D33	3	5
D34	3.5	5
D35	3	5
D36	3	4
D37	3	7
D38	3.5	5
D39	4	5
D40	3	6
**Median**	**4**	**5**
**Q1**	**3**	**5**
**Q2**	**4**	**6**
**IQR**	**1**	**1**

Table S7. Dogs' body and muscle condition score.

Q1- the first quartile, Q3- the third quartile, IQR- interquartile range
